# Insulin‐like growth factors and their potential role in cardiac epigenetics

**DOI:** 10.1111/jcmm.12845

**Published:** 2016-04-07

**Authors:** Cristiana Iosef Husted, Maria Valencik

**Affiliations:** ^1^ Department of PharmacologyUniversity of Nevada, Reno School of Medicine (UNSOM)RenoNVUSA

**Keywords:** transgenerational epigenetics, CVD, IGF, chromatin‐memory

## Abstract

Cardiovascular disease (CVD) constitutes a major public health threat worldwide, accounting for 17.3 million deaths annually. Heart disease and stroke account for the majority of healthcare costs in the developed world. While much has been accomplished in understanding the pathophysiology, molecular biology and genetics underlying the diagnosis and treatment of CVD, we know less about the role of epigenetics and their molecular determinants. The impact of environmental changes and epigenetics in CVD is now emerging as critically important in understanding the origin of disease and the development of new therapeutic approaches to prevention and treatment. This review focuses on the emerging role of epigenetics mediated by insulin like‐growth factors‐I and ‐II in major CVDs such as heart failure, cardiac hypertrophy and diabetes.

## Introduction

Epigenetics is an emerging frontier of science that involves the study of gene regulation when the DNA‐primary structure remains intact. While epigenetics refers to single genes or sets of genes, epigenomics denotes the global analyses of epigenetic changes across an entire genome 1, 2. In the early 1940s, Waddington described epigenetics as ‘the interactions of genes with their environment, which bring the phenotype into being’. Phenotype inheritance is governed by genetics, whereas epigenetic modifications are acquired during life and largely reset between generations, especially at the time of conception 3. The modifications that bypass this reset are referred to as ‘Transgenerational Epigenetics’ 4.

Factors that induce epigenetic modifications can be *transient or permanent* and it is difficult to detect or associate them with a certain phenotype. Epigenetic changes can be elicited by metabolic alterations, environmental stress, toxicants, viruses and immunity 5, 6, 7, 8. The principal mechanisms underlying epigenetic inheritance are governed by DNA methylation, histone modification and the action of non‐coding RNAs (ncRNAs) 9, 10, 11, 12, 13. Even the most stable epigenetic mark which is the DNA methylation still displays plasticity during development and ageing 14, 15, 16. Furthermore, in early life, transient exposures to unbalanced nutrition, endocrine abnormalities, low oxygen tension, intense exercise, environmental pollutants or increasing age can alter DNA methylation with severe health impacts including cardiovascular disease (CVD) 17, 18, 19, 20, 21, 22, 23. Ongoing research is expected to clarify whether induced phenotypic traits among multiple generations operate through altered epigenetic marks in germ cells and/or through *de novo* formation of epigenetic marks in each generation. Most of the DNA methylation marks (expressed as hypo‐ or hypermethylation clusters) are cleared during fertilization and only certain characters are someway preserved 24. This suggests a potential mechanism of transgenerational inheritance that denotes a process whereby treatments or insults experienced by the gestating mother (F0) and the developing foetus (F1) may cause epigenetic changes (‘paramutations’ or ‘marks’) that are propagated to the (F3) generation through the gametes of the affected foetus, which was not exposed to the initiating conditions 25, 26, 27, 28, 29. Such inheritable traits are not spontaneous and they appear after exposures to certain environmental factors, bypassing the global epigenetic reprogramming during embryogenesis, when the epigenetic clearance process mandates a reset of the entire parental profile. Inherited, self‐propagating epigenetic marks depend on the timing of their onset, the chemical nature of the modification, the DNA‐sequence affected, the number of copies and finally their location (site specificity). Transgenerational epigenetic inheritance was first observed in classical transgenic mouse models where constitutively active and randomly integrated transgenes, regardless of the numbers of copies, became heritably silenced *via* cytosine‐phospo‐guanine (CpG) methylation, when the germline was challenged by mating with a non‐transgenic phenotype 30. In inducible transgenic models, the genes and their site‐specific integration can be precisely controlled. Thus far, it is known that single generation epigenetic marks depend on the timing of their onset, while their transgenerational inheritance depends on their location (loci). Both repressive and activating chromatin modifications can be transgenerationally inherited and their investigation has been significantly improved by the Cas‐9‐based editing method 27.

Epigenetics represents crucial regulatory mechanisms in evolution, development, ageing, adaptation and disease. Therefore, making correlations between environmental exposure, epigenetic marks and disease should be of high priority in the near future. It is important to elucidate how biological systems adapt or not to environmental changes. The dynamics of epigenetic characters show that organisms respond either reversibly or irreversibly to environmental signals and that modifies tissue‐ and cell‐type‐specific gene programmes. During ontogenesis, epigenetic marks are associated with high biochemical plasticity. Growth factors, cytokines and adhesion molecules govern multiple cellular processes in response to microenvironmental changes during development. To date, little is known about the extracellular signalling and the mechanisms that allow adaptation in response to the changes in microenvironment. Among all, growth factors can be critical determinants of both the onset and progress of the epigenetic modifications. For example, insulin like‐growth factor (IGF I) can act in both systemic‐ and tissue‐specific manners altering both metabolic and non‐metabolic signalling pathways that can induce epigenetic changes for the entire life span of an organism 31, 32, 33. There are two IGF‐molecules (IGF‐I and ‐II) and they are both epigenetically regulated (Table 1). Each organ or biological event has specific histone and DNA‐methylation codes as well as specific prerequisites for growth factors. Effectors such as histones, micro‐RNAs or different regulators of transcription are largely unknown in epigenetic pathology. Thus, in Table 1, we summarized information that suggests possible epigenetic codes for several physiologic and physiopathologic entities that are related to IGF‐signalling. From this perspective, a few questions will soon need answers: (*i*) Are the epigenetic changes preserved at key loci *via* germ lines? If yes, how would IGF‐fluctuations affect them? (*ii*) Is their maintenance based on the magnitude and/or the timing of the IGF‐stimuli? (*iii*) Are the epigenetic marks erased and/or reset in the presence or absence of IGFs? and (*iv*) What are the mechanisms responsible for the formation of epigenetic marks and what role do IGFs have in this process?

**Table 1:** Epigenetic regulation of insulin‐like growth factors, IGF‐I and IGF–II

IGF molecules	Epigenetic regulation			Regulations of transcription	Associated physiopathology or physiology	References
	Histone code	Methylated markers	Micro‐RNAs			
IGF‐1	Histone 3me	Methyl CpG binding protein 2	Let7f		Rhet syndrome	41, 42
Epigenetically regulated	H3K4me3/H3K9me3	b‐Myosine Heavy Chain	miR‐1, miR‐133	PAX3/FOXO1, NFkB	Cardiac hypertrophy	39, 48, 69
	HDAC4		mir‐1/206, miR29, miR‐26a	miR26a/EZH2; miR‐29/YY1	Muscle development	32, 48
				PAX3, PAX7, SRF/MEF2, Wnt		
	H4K12ac		miR‐98		Alzheimer's diseases	40
	me3K36, me2K4	DOL0, DOL21	miR‐210,‐144,‐451,‐146b‐5p,‐126,16,‐29b,‐26b,‐335,‐182,‐155,‐20a		Intra‐Unterine‐Growth‐Restriction (IUGR)	36, 43, 49
	Not known	PAX5, GATA‐2, ARHGAP5	miR‐486		Lung cancer	37, 40
IGF‐2	H3K9me2	H19 (murin models)	miR‐30E		Embryonic development	35, 44
Imprinted gene		CTCF binding sites	Not known		Placental growth	45
Epigenetically regulated	HDAC4		miR‐125b, LIN‐28	Myf5	Inhibits muscle differentiation	32, 38
			miR‐1275, miR‐483‐3p		Cancer	46, 47

## Implications of IGFs in cardiac epigenetics

**Figure 1:**
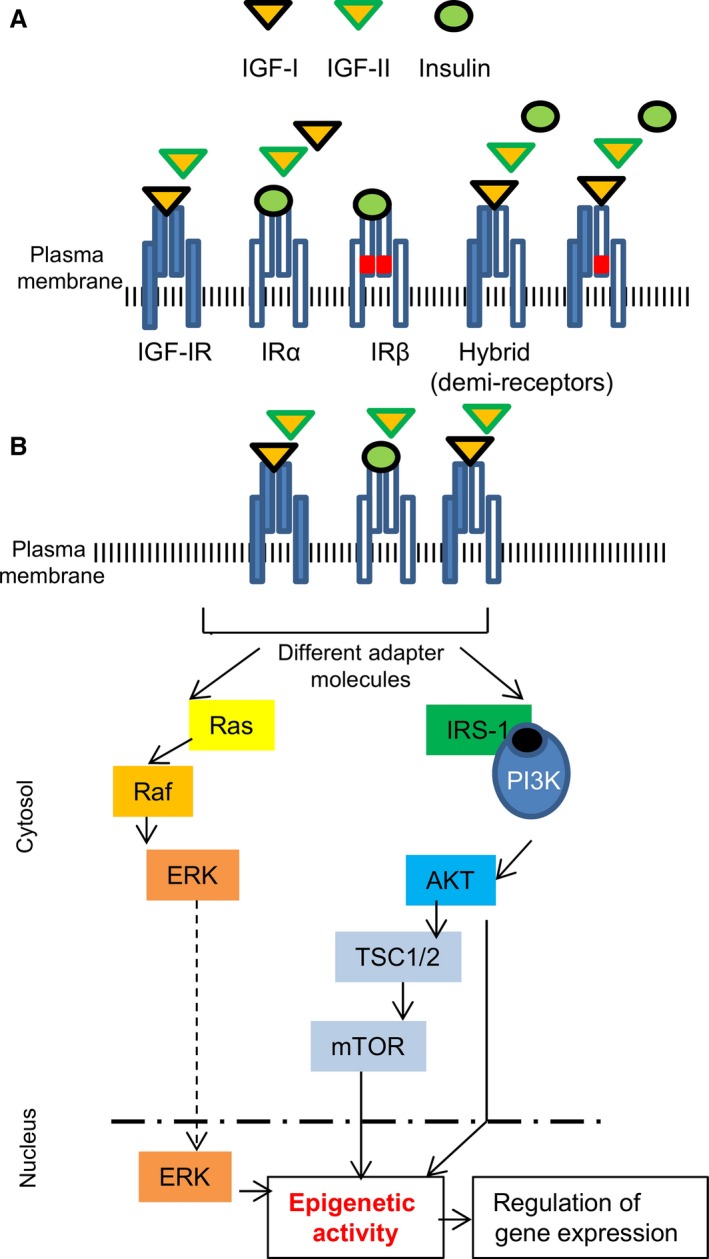
Implications of the IGF system in epigenetics. (**A**) Schematic representation of the receptors and ligands underlying epigenetic change. Insulin receptor isoforms (IR‐α or IR‐β) bind insulin with high affinity, while IGF‐I receptor (IGF‐IR) binds IGF‐I and IGF‐II. In cells expressing both IR and IGF‐IR, IR may heterodimerize with IGF‐IR receptors, leading to the formation of hybrid (demi) receptors such as IR/IGF‐IR (HRs). HRs bind IGF‐I and IGF‐II with high affinity and insulin with low affinity. (**B**) Diagram of the two major signalling pathways of the IR and/or IGF‐IR and their hybrid receptors (demi‐receptors). ERK and mTOR are potential candidates for initiation of epigenetic activity and subsequent regulation of gene expression.

The IGF system is comprised of five related receptors, three ligands (Insulin, IGF‐I and ‐II) and six IGF‐binding proteins (IGFBPs) 50, 51, 52 (Fig. 1A). The type‐I IGF receptor (IGF‐IR) and the insulin receptor (IR) are tetrameric glycoproteins that have two extracellular and two transmembrane subunits linked by disulfide bonds. The extracellular subunits contain the ligand binding site, whereas the carboxy‐terminus transmembrane subunits have tyrosine kinase (TK) domains. As RTKs, IGF‐IR and IR are overall more than 50% homologous and share 84% resemblance in their TK domains; therefore, they can signal through similar pathways and compensate for each other's functions. In addition, for hybrid receptors which bind both insulin and IGF‐I, the transmembrane subunits play a decisive role in recruiting intracellular mediators through their Tyr residues. This is common in muscle progenitors but also in fully differentiated cells 53, 54, 55, 56, 57 (Fig. 1B).

### How important are IGF‐IR and IR to cardiac biology?

There are a few signalling mechanisms triggered by ligand‐binding to IGF‐IR and IR that have the potential to mediate skeletal and cardiac muscle epigenetics. These pathways are driven by the activation of PI3K (Phosphatidylinositol‐4,5‐bisphosphate 3‐kinase) and AKT (protein kinase B). (*i*) One target of this signalling cascade can be the mTOR molecule (mammalian target of rapamycin). Insulin like‐growth factors‐I, ‐II and insulin bind to their receptors by activating the PI3K/AKT/mTOR pathway and the Ras/Raf‐1/MEK/ERK signalling cascade 58, 59. *Nutrient‐sensitive PI3K–AKT–mTOR* pathways have a crucial role in regulating the balance between quiescence and proliferation of progenitor cells in many tissues including the heart muscle 60, 61, 62. The mTOR complex integrates the input from upstream pathways, including insulin and IGFs. mTOR also senses cellular nutrient, oxygen, and energy levels and it has key‐regulatory function in cardiovascular physiology and pathology being a good candidate for epigenetic regulation. The mTOR complex is the catalytic subunit of two structurally distinct molecular entities: mTORC1 and mTORC2. In general, mTORC1 regulates protein synthesis, growth and proliferation, autophagy, metabolism and stress responses (being required for cardiovascular development and postnatal maintenance). In addition, mTORC1 is necessary for cardiac adaptation to pressure overload and the onset of the compensatory hypertrophy. By contrast, mTORC2 seems to regulate cell survival and cell polarity. Thus, malfunction of the IGF‐system can potentially alter the heart organ maintenance through the PI3K/AKT/mTOR signalling loop. Multiple actions of IGF‐I have been described in both cardiac and striated muscle cells, including the well‐documented anti‐apoptotic effect and the newly emerged action on regeneration 63, 64.

(*ii*) Another mechanism led by the IGF‐I and AKT activation is related to the functions of miR‐1, a muscle regulator, lately found to be responsible for transgenerational cardiac hypertrophy in mice 65. When AKT is overexpressed, IGF‐I controls miR‐1 levels through Foxo3a transcription factor 69, 92, 93. The reciprocal relationship between IGF‐I and miR‐1 indicates a great potential role of the IGF‐I axis in CVD‐related epigenetics 94, 95. The interplay between IGF‐I, AKT, Foxo3a and miR‐1 suggests a new paradigm that emphasizes the fashion in which IGF‐I may regulate cardiac and skeletal muscle epigenetics transgenerationally. In support of the mouse model are several reports describing humans with acromegaly, a syndrome caused by GH/IGF‐I overexpression that induces cardiac hypertrophy associated with low miR‐1 levels 69, 92, 93. These effects may be explained by IGF involvement with chromatin memory formation where IGF‐I activates AKT, and in turn, AKT regulates enhancer‐zheste‐homologue‐2 (EZH2), a polycomb‐group protein that was lately defined as critical in chromatin repression, especially in progenitor cells. Given this, we suggest that IGF‐I is a strong mediator for epigenetic mark formation where micro‐RNAs such as miR‐1 can be important players 59, 60, 61, 62, 63, 64, 65, 66, 67, 68, 69, 70, 71, 72, 73. From this perspective, it is safe to hypothesize that the miR‐1‐induced epigenetic marks that allow cardiac hypertrophy may be, in fact, IGF‐I‐dependent.

(*iii*) The IGF‐I/AKT pathway is also involved with sugar and fat metabolism. It is known that IGF‐I, insulin and the glycolytic network regulates the development of skeletal and cardiac muscle, including progenitor cells 31, 74, 75. Production of IGF‐I is stimulated by GH and it can be modulated by different environmental factors, especially stress and nutritional insults that have been shown to have great potential for epigenetic disease. Animal models should be very useful to explore these features in the heart, although IGFs are difficult to manipulate in human or animal subjects because of their temporal‐ and dose‐dependency. Moderate concentrations of IGF‐I may stimulate AKT and drive adaptive/physiological hypertrophy. On the other hand, excessive levels of IGF‐stimulated p38‐mitogen‐activated protein kinase (MAPK) are limiting cardiac hypertrophy through myostatin production, negatively regulating progenitor cell differentiation 76. (*iv*) Both GH and IGF‐I are widely used as performance enhancing drugs in adult or paediatric medicine and little is known about their epigenetic implications in short or long term. Because of the hypertrophic effects observed in both skeletal and heart muscle under intensive IGF‐I treatments, it is mandatory that its epigenetic action should be thoroughly analysed. We suggest that future studies should actually include the entire ‘GH/IGF axis’, which can potentially be responsible for the regulation of histones H3 and H4 acetylation and the activation of chromatin repressors such as EZH2. All these effects would still be under the action of the IGF‐I/AKT signalling loop regardless of the initial trigger 31, 35, 76, 77.

### How important are the interactions between IGF‐IR and other receptor‐TK (RTKs)?

Many developmental growth factors such as IGF‐I, insulin, vascular endothelial growth factor (VEGF) and stromal growth factor signal through RTKs. One event that can emphasize the interference between two important RTKs in heart physiology is ischemia/reperfusion (I‐R) 77. It is highly probable that this process initiates epigenetic effects. In I‐R, the expression of VEGF is regulated by hypoxia and cytokines but also by IGF‐I by reciprocity. In isolated hemo‐perfused pig hearts with acute ischaemia, RTKs such as IGF‐IR and VEGF‐R along with foetal liver kinase (flk‐1) or fms‐like tyrosine kinase‐1 (flt‐1) were significantly increased in comparison to normal hearts 77. Such data certainly suggest a possible cross‐talk between VEGF and IGF‐I and their signalling partners during the initiation of CVD.

Cross‐talking between RTKs is probably caused because different ligands are steering the activation of key molecules such as AKT. In this context, AKT can act on glycogen synthase kinase 3 (GSK3), which is a Ser/Thr kinase with important role in the regulation of an important developmental loop led by Wnt signalling and glycogen synthesis 78. Glycogen synthase kinase 3 is inactivated by AKT and this leads to glycogen synthesis and muscle development as a crucial process in CVD. Contribution of Wnt‐mediated activation of β‐catenin has been observed during skeletal development and reported by a large number of studies, but the role of the IGF‐I/AKT action on β‐catenin is still not clear, although some reports show that IGF‐I can nevertheless lead to active β‐catenin 78. The inactivation of GSK3/Wnt signalling loop is critical for embryonic development, including skeletal and heart muscle. Once these mechanisms will be established in the future, they will become tremendously important to define the epigenetic basis of the CVD onset.

## Can IGFs induce or reset epigenetic traits?

Development is controlled through a temporal and spatial sequence of events including both activation and silencing of specific genes. Taken together, these events contribute to genetic reprogramming and they can be altered by environmental conditions that induce chromatin remodeling and/or ‘transcriptional memory’. A classic example in this sense is the ‘fertilized egg cell’, which represents a checkpoint in evolution because at this stage, most of the epigenetic modifications acquired by the parental lines are cleared, whereas a small proportion of the traits are transferred across the generations 79, 80, 81.

Nonetheless, the power of the current genomic technologies cannot yet analyse in‐depth the causes of the epidemiologic determinants that govern the hereditary pathologies of non‐Mendelian origin (such as many CVD cases). The ‘non‐Mendelian’ types of heredity result in transmission of epigenetic features and toning of gene expression. The first observations in mammals have been made by Dr Minoo Rassouzadegan who showed for first time that non‐Mendelian inheritance of an epigenetic colour variation can be reproduced in mice 66. The study was subsequently extended to a case of cardiac hypertrophy 65, 69, and then to a remarkable variation in embryonic overall growth 67. In all three cases, the determinant epigenetic controls were exerted by microRNAs injected into mouse fertilized eggs. These studies could be of course furtherly extended in stem cell or animal breeding technologies. Inspired by these findings, our projects (Iosef Laboratory, University of Nevada) are focused on decrypting the molecular mechanisms that insure the docking of the exogenous micro‐RNAs to the selected mRNA targets in the fertilized eggs. It is known that in the nature there are four distinct types of RNA molecules that coordinate gene expression in all cells: mRNAs, ribosomal RNA, transport RNA and finally small non‐coding RNAs (snRNAs) which are highly conserved and particularly abundant in the nuclei of mammalian cells where they are tightly bound to one or more proteins in particles called small nuclear ribonucleoproteins. Small regulatory RNAs of only 22 nucleotides long such as micro‐RNAs have been also identified in creating micro‐RNPs corpuscles 68. Thus, such complexes became the center of our attention in the context of studying the role of the micro‐RNAs in determining the inheritable epigenetic events in fertilized egg cells. We believe that micro‐RNPs control both the translation and mRNA stability allowing epigenetic functions. One example can be miR‐1, which injected into mouse fertilized eggs induced cardiac hypertrophy and slightly reduced IGF I expression. We hypothesize that miRNPs are actually factors that help micro‐RNAs in selecting the right mRNA targets for the right cell fate according to the microenvironmental requirements. This hypothesis is perfectly supported by findings published by the Steintz group (Yale) 68, which are showing that miRNPs function can indeed dock snRNAs but this action is dependent on the cell cycle stage and the presence of certain growth factors 68.

### What is the basis for these mechanisms?

The answer is very complex. Normal and pathologic epigenetic mechanisms are based on signalling and chromatin memory. The diseased state is generated by abnormal signals, most induced by the cellular microenvironment. These signals are translated into phosphoregulatory events that precede alteration of chromatin stability, ultimately shifting gene expression. Chromatin assembly is still largely unexplored in developing tissues where phenotypic plasticity is high and signals sent by growth factors such as IGFs are critical. In addition to development, during ageing, phenotypic plasticity can be also regulated by growth factors, cytokines and extracellular matrix fluctuations leading to new epigenetic profiles. The questions that we suggest for future studies refer to the role and mechanisms of IGF‐mediated epigenetic remodelling in both development and ageing. In general, gene repression is mediated by a specific sequence of events including recruitment of polycomb group proteins (PcG), histone modifications and DNA methylation.

#### IGFs and PcG recruitment

**Figure 2:**
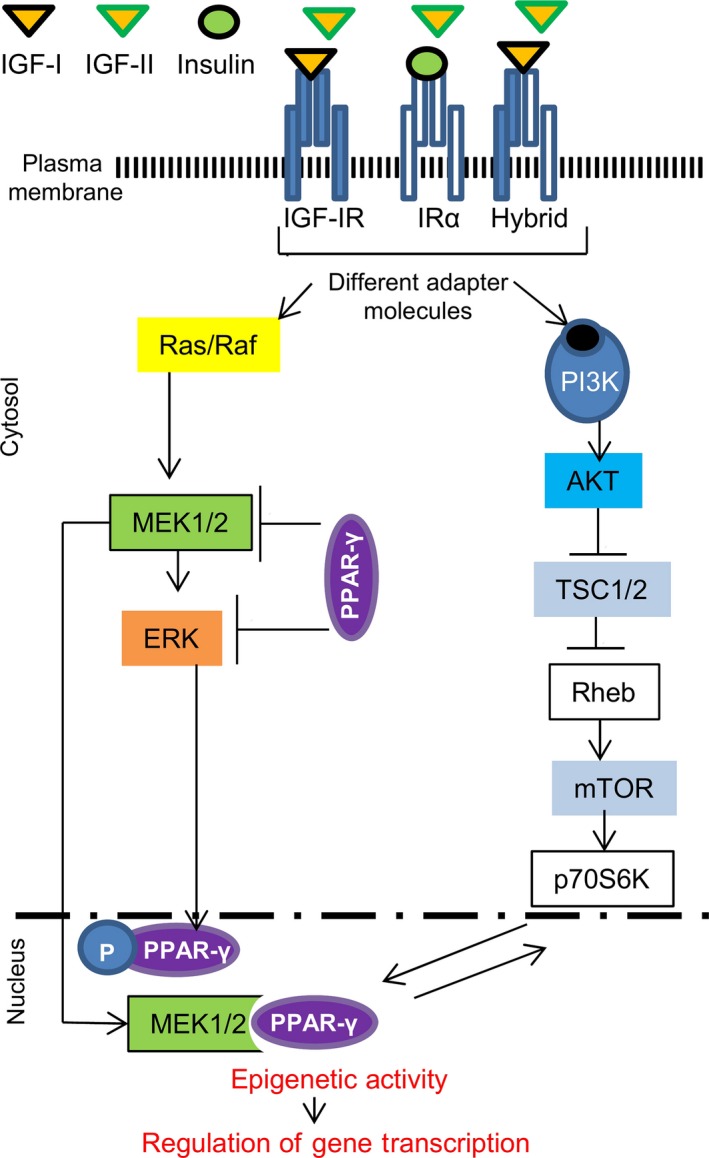
Proposed IGF‐interaction with PPAR‐γ signalling in CVD epigenetics. Interaction between PPAR‐γ and MAPK/PI3K pathways happens at different levels or time points in the cell cycle, thus in certain situations PPAR‐γ reduces the expression of MEK1/2 protein expression and inhibits ERK1/2 phosphorylation 60, 146, 148. In contrast, in other cell systems, PPAR‐γ may activate ERK1/2. Furthermore, the interaction between PPAR‐γ and MAPK/PI3K may include pathways where ERK mediates PPAR‐γ phosphorylation, MEK1/2‐dependent PPAR‐γ nuclear export followed by PPAR‐γ degradation, PI3K inhibition through PTEN, mTOR decrease by AMPK activation and finally p70S6K phosphorylation. This signalling loop can be potentially responsible for epigenetic activity that targets survival, proliferation and/or differentiation of cardiac cells. Physical effort has been shown to increase levels of IGF‐I in muscle and physiological cardiac hypertrophy, thus inducing the IGF‐I/PI3K/Akt/P70S6K signalling pathway, and thereby increasing the protein synthesis required to build muscle; all of this can be epigenetically regulated 34, 60, 153, 154. On the other hand, PPAR‐γ ligands (such as rosiglitazone) activate tuberous sclerosis complex‐2 (TSC2) inhibiting mTOR signalling 152, 155 as a possible compensatory effect to the MEK/ERK action on PPAR‐γ.

**Figure 3:**
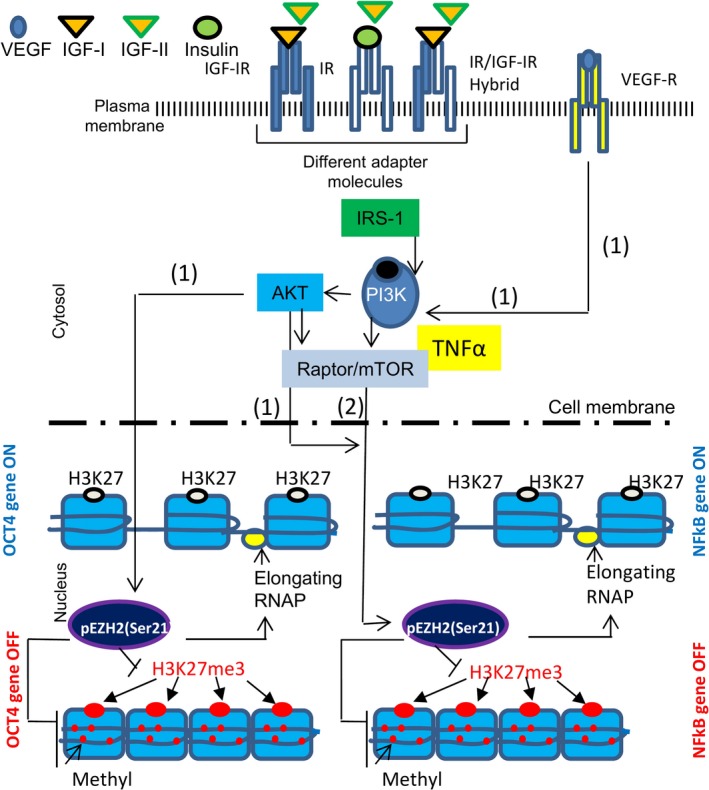
Potential epigenetic actions of IGF‐I in the developing heart muscle. When histone 3 (H3) is tri‐methylated (me3) at lysine 27 (K27) residue, it is associated with *inactive* gene promoters. Because of its dramatic and predictable effect on gene expression, H3K27me3 is a great marker for epigenetically inactivated genes. EZH2 catalyzes di‐ and tri‐methylation of the K27 residue of histone H3 (H3K27me2/3). High expression of EZH2 in stem cells is associated with modified nucleosomes at the promoters of important developmental transcription factors such as OCT4, thus preventing premature activation of the lineage‐committing markers 158, 159. Growth factors such as IGFs activate AKT that in turn phosphorylates EZH2 at Ser^21^ residue blocking its methy‐transferase activity and consequently de‐repressing gene‐promoters. (1) Based on the above principles, here we hypothesize that IGF‐I and/or vascular endothelial factor (VEGF) can activate AKT which in turn induces pEZH2^Ser‐21^ suppressing its methyltransferase activity and the binding of histone H3 at the OCT4 promoter 79 in cardiac stem cells. (2) It is known that EZH2 and NFkB crosstalk in cancer mechanisms 84 where EZH2 functions as a multitasking molecule that can either act as a transcriptional activator or a repressor of the NF‐κB targets 84. This will depend on the cellular micro‐environment and perhaps on IGF‐I that may be a key‐factor. In fact, IGF‐I can signal either through AKT or through the IGFIR/PI3K/Raptor/mTOR loop and in both cases it is possible that it may de‐repress the NFkB promoter through EZH2 157. Ablation of NFkB activity with a specific inhibitor (BAY) impairs vasculogenesis in the neonatal lung and in the same tissue chromatin immuno‐precipitation reactions showed that NFkB binds directly the promoter of the VEGF‐2 59. Here we hypothesize that either VEGF or IGF‐I signalling through AKT, activates mTOR which in turn may lead EZH2 polycomb protein to de‐repressing the NFkB promoter, making possible the remodeling of the endothelial tissue in the heart.

Once bound to its receptor, IGF‐IR, IGF‐I ligand triggers phosphorylation events that activate AKT/PKB signalling loop and this targets the function of PcGs 34. Polycomb group proteins are highly conserved constituents of evolutionary molecular pathways that regulate cell fate (Fig. 2 and Fig. 3). They form two multimeric protein complexes (PRC‐1 and ‐2) that are involved in the inheritable‐stable repression of genes because of the modification of chromatin structure 34, 82. Both PRC‐1 and PRC‐2 can be regulated by RNAi or non‐coding RNAs 83. The main effectors of the polycomb repressive complex (PRC2) are embryonic ectoderm development protein and EZH2 79, 156, 158, 159. They act early in development to set the stage for the long‐term repressive PRC1 complex 158, 159. The PRC2 complex specifically methylates histone H3 at lysine 27 (H3‐K27), providing an epigenetic mark for binding PRC1 complex. Enhancer‐zheste‐homologue 2 protein within the PRC2 complexes can methylate Lys 9 (H3‐K9) and Lys 27 (H3‐K27) of histone H3 together with Lys 26 of histone H1 (H1‐K26). Therefore, EZH2 activity may be considered a landmark for the formation of epigenetic marks in developing tissues as they are mediated by the growth factors and AKT actions 34, 158, 159. But how does it happen? Specifically, phosphorylation of Thre^350^ has been linked to an *increase* in EZH2 activity while activation of Thre^492^ and Ser^21^ has been linked to a *decrease* in its activity. AKT phosphorylates EZH2 at Ser^21^ and overturns its methyltransferase activity by obstructing its binding to histone H3 84. This results in a decrease in Lys^27^ tri‐methylation in H3 and de‐repression of silenced genes. In this way, AKT regulates the DNA methylation by acting on EZH2 and this may further contribute to development 34. Our labarotory is currently exploring the activity of EZH2 at the promoters of genes that encode important developmental transcription factors such as OCT‐4, as initiated by AKT post‐IGF‐IR‐activation in muscle progenitor cells (Fig. 3). We are also researching the possibility where NFkB becomes a developmental factor implicated in endothelial formation as triggered by IGF‐I and VEGF signalling. It is known that EZH2 and NFkB crosstalk in cancer mechanisms 84. For example, EZH2 functions as a multitasking molecule in breast cancers, where it can either be a transcriptional activator or a repressor of the NF‐κB targets depending on the cellular micro‐environment (where IGF‐I may be a key‐factor) 84. We assume that IGF‐I can signal through the IGFIR/PI3K/Raptor/mTOR loop that may possibly de‐repress the NFkB promoter through EZH2. Based on the above hypothesis, we investigated the role of IGF‐I in transcription of the OCT4 gene which directly changes stem cell fate 85 (Fig. 3). Our preliminary (unpublished) studies show that IGF‐I but not IGF‐II mediates the binding of the EZH2 repressor to the promoter of the gene encoding the OCT4 transcription factor 85. This study may contribute in the future to a better understanding of the role of IGF‐I in stem cell maintenance and differentiation.

#### IGFs and histones

Insulin like‐growth factors are implicated in many post‐translational modifications mediated by histones or in those that directly affect histone activity. For example, butyrate‐induced IGF‐II activation is correlated with distinct chromatin signatures because of histone modification. Chromatin immunoprecipitation assay indicated that association of acetyl‐histone H3 and acetyl‐histone H4 with the IGF‐IIR promoter was increased in the presence of ANGII. This study demonstrated that histone acetylation plays a critical role in IGF‐IIR upregulation during cardiac diseases and it might provide in the future a real option for targeted gene therapy in heart failure 91. Another example of epigenetics governed by histones and IGFs is intrauterine growth restriction (IUGR), where a decrease in postnatal IGF‐I mRNA variants is associated with histone 3 tri‐methylation of lysine 36 at the *igf1* gene (H3Me3K36). Even in this case, IGF‐I‐dependent changes in histone H3 modifications are associated with AKT signalling and the expression of cell survival genes 86.

In addition to the IGF‐ligands, IGFBPs too are related to histone biology. For example, the expression of IGFBP‐3 is frequently suppressed in liver cancers and it can be reactivated by histone deacetylase (HDAC) inhibition 51, 87, 88, 89, 90. IGFBP‐1 also has epigenetic effects through the induction of cAMP, which results in the alterations of histone acetylation and retargeting in human endometrial stromal cells. Another IGF‐regulatory protein, IGFBP‐6, was also reported to potentially interact with histones. In this case, IGFBP‐6 and H2B seem to be involved in the development, ageing and neoplastic transformation of muscle tissues 68, 69.

#### IGFs and DNA methylation

DNA methylation is a covalent modification that occurs in mammals predominantly at cytosines followed by guanines (CpG dinucleotides) to form 5‐methylcytosines. Cytosine‐phospo‐guanine methylation is commonly associated with gene silencing. DNA methylation status has fluctuations during lifetime and has been shown to regulate biological processes underlying CVD features such as atherosclerosis, inflammation, hypertension and diabetes. Indirectly through its actions, IGFs may contribute to changes in DNA methylation.

## Can IGFs control cardiac epigenetic traits induced by nutritional insult?

Cardiovascular disease remains the leading cause of death worldwide despite the efforts made to reduce its incidence 96, 97. One explanation could be the multifactorial origin of this illness and the massive distribution of the environmental conditions that may alter the genetic 98, 99, 100 and epigenetic programming of the cardiovascular system 14, 15, 94, 95, 101, 102. Unfavourable lifestyle factors and diseases such as dyslipidemia, diabetes 101, 103, obesity 104, 105, hypertension, physical inactivity, alcohol consumption and cigarette smoking 106 are the main risk elements for CVD. However, a smaller but significant fraction of patients develop CVD in the absence of traditional risk factors, and evidence indicates that this type of CVD may become an inherited disease of non‐genomic origin. Therefore, the epigenetic contribution of the risk factors and their role in CVD predisposition are important research targets and IGFs may become one of them in the near future 107, 108, 109, 110, 111, 112, 113, 114, 115, 116.

### IGFs as epigenetic determinants of nutrition‐related pathologies involved with CVD

The epigenetic modifications in the foetus can have maternal origin (pregnancy associated challenges) or they can be originated in the foetus itself. Development depends mainly on the maternal health with nutrition as a major determinant 109, 110. In particular, maternal malnutrition impairs foetal development, resulting in a small placenta and low birth weight. This process is IGF‐dependent and predicts chronic disease, including CVD in the adults. Inappropriate nutrition targets one‐third of the world's population and out of this fraction, ~50% is submitted to obesity—which is the promoter of many degenerative diseases and particularly CVD. Actually, maternal *obesity* leads to a specific phenotype, namely ‘Large for Gestational Age (LGA)’ *Syndrome* which is a major risk factor for obesity, diabetes and even cancer in the offspring 110, 111. Remarkably, *malnutrition* can promote similar effects during foetal development. A classic example in this sense is the population born from mothers exposed to the *Dutch Hunger Winter at the end of World War II* (1944–1945). In this group, another syndrome has been identified, namely ‘Small for Gestational Age (SGA)’ 118, 119. Infants with SGA show a greater occurrence of impaired glucose tolerance, obesity, hyperlipidemia, hypertension, micro‐albuminuria, sometimes psychiatric disorders and a greater mortality rate because of CVD. However, the SGA phenotype allows a better survival rate if starvation continues after birth compared to the situation when the offspring is overfed. Both LGA and SGA phenotypes favour fat storage and diabetes (with insulin resistance) if overfed later in their post‐natal life. Large for Gestational Age and SGA phenotypes are atleast partially caused by the malfunction of the IGF/Insulin systems. Added to this is the fact that under nutritional insult, IGFs may interact with the action of certain micro‐RNAs. Some of these miRNAs with altered expression are involved in the regulation of mTOR, insulin, adipo‐cytokines 120, 121, 122, leptin 120 and MAPK. These pathways that are mainly led by the malfunction of the IGF system may contribute to the onset of the metabolic dysfunctions responsible for CVD in the progeny, which can be propagated in a *transgenerational manner*. Another aspect of the IGF‐induced epigenetics based on nutritional insult is the bio‐activity within the foetal microenvironment. This may drive transgenerational effects founded on specific genomic and/or epigenomic settings that can be rearranged according to the intensity and duration of the IGF fluctuations 108. This is important because foetal development involves numerous morphogenetic processes associated with cell growth, proliferation and differentiation that are greatly influenced by IGFs 81 and factors such as nutrition can interfere or induce foetal reprograming 110. Nutritional insults during pregnancy can either be caused by excessive or insufficient maternal food 111, 112, 113. Either way, the food intake can alter the metabolic control in the offspring increasing the risk for metabolic diseases and indirectly leading to CVD 110, 114, 115, 116.

In addition to global nutrition, single micronutrient deficiency has a transgenerational impact on the offspring that can also result in dramatic effects 123, 124, 125, 126. For instance, dietary zinc (Zn) restriction during pregnancy induces low birth weight in the newborn rats, hypertension, kidney failure and endothelial dysfunction in adult rats 127. All these features show a predisposition to CVD that can be potentially related to the biology of the IGF system. Specifically, IGF‐I and IGFBP‐3 serum levels are low in children with Zn deficiency, and increased after Zn supplementation for 3 months 128.

### Deficient protein metabolism, IGFs and CVD

Red meat such as lamb, beef and pork may induce CVD through yet unidentified mechanisms associated with high levels of *methionine* 129. It has been suggested that a diet rich in methionine can induce CVD by oxidative stress, inflammatory manifestations and vascular remodeling. Mice fed with methionine developed CVD with LV dysfunction marked by higher levels of superoxide dismutase‐1 in the hearts, while interleukin (IL)‐1, IL‐6, tumour necrosis factor α and TLR4 (toll‐like receptor 4) showed a significant inflammatory profile 62, 129. The upregulated levels of eNOS/iNOS and MMP2/MMP9 beside high collagen deposition indicated vascular remodeling in same methionine fed mouse model 129. Thus, high methionine levels are considered to be a CVD hazard increasing the oxidative stress, inflammatory manifestations and matrix/vascular remodelling finally altering the cardiac function. These mechanisms are not yet known to interfere with IGFs actions but they are open for further studies.

Another example based on protein metabolism is given by the high levels of the plasma *homocysteine* that are associated with insulin resistance syndrome and this might involve IGFs actions 130, 131, 132. This stressor represents a risk factor of epigenetic origins for CVD. It has been previously shown that genome‐wide methylation—as determined by investigation of LINE‐1 sequences (as marker) and CpG dinucleotides within consensus coding sequence genes—were all associated with high levels of homocysteine 130, 131, 132. In fact, homocysteine impairs placental amino‐acid transport producing restricted foetal growth and possibly CVD by epigenetic mechanisms that may interfere with insulin and IGFs signalling.

### Deficient lipid metabolism, IGFs and CVD

Various examples emphasizing the IGFs action in modulating the CVD epigenetic origins can be given by different lipid metabolism situations 133, 134, 135, 136, 137, 138, 139, 140, 141, 142, 143. Among many, the biology of the peroxisome proliferator‐activated receptors (PPARγ and PPARα) is potentially related to IGFs and this is emerging as a possible interface between lipid metabolism, epigenetic modifications, atherosclerosis and CVD 144, 145, 146, 147, 148. (*i*) Several target genes of PPARγ transcription factors have been identified, including GATA2 or Krüppel‐like factor family 149. PPARγ simultaneously regulates Setdb1 and Setd8 150, two histone lysine methyl transferases that cause chromatin modifications which lead to adipocyte differentiation. Furthermore, as presented by Mur *et al*. in their 2003 report 151, IGFs have an important role in insulin‐induced brown adipocyte differentiation through PPAR‐γ. PPAR transcription factors can potentially become markers for assessing CVD risk in relation to insulin and IGFs abnormal levels. Lecka‐Czernik *et al*. 152 demonstrated that activation of PPAR‐γ by its ligand (rosiglitazone) suppresses components of the IGF regulatory system *in vitro* and *in vivo* (Fig.2). Developing novel adipocyte‐specific PPARγ activators or setting up a complex therapy including thiazolidinediones and epigenetic modulators may selectively regulate the promoters of adipogenic genes, henceforth, fulfilling important therapeutic purposes including the treatment of CVD 153. (*ii*) The PPAR genetic profiling uncovers *igf‐1* gene as a *PPAR‐α* target with an important role in cardio‐protection. PPARα activation in the wild‐type mouse heart resulted in up‐regulation of the *igf‐1* gene transcription and provided protection against cardiomyocyte apoptosis post ischemia or biomechanical stress. This confirms that *igf‐1* gene is an *in vivo* target of PPARα and the involvement of a PPARα/IGF‐I signalling loop in cardiomyocytes protection under ischemic and hemodynamic loading conditions is a must 154. In other cases, such as parental exposure to high‐fat‐diet or obesity in F2‐generation of mouse descendants has been associated with *PPARα* as the main molecular player in epigenetic transgenerational inheritance 142. In addition, these epigenetic modifications were associated not only with an increase in body size but also the reduction of insulin sensitivity which may cause IGF‐malfunction and subsequently CVD.

It should be noted that IGFs do not act alone within the PPAR circuit but in strict relationship with IGF‐binding proteins. For example, between IGF‐I and insulin there is a feedback mechanism ensured by IGFBP‐1 155. IGFBP‐1 modulates IGF‐bio‐availability for glucose homeostasis and it is inhibited transcriptionally by insulin. Treatments with the anti‐diabetic troglitazone have affected IGFBP‐1 production and the activity of PPARα and PPARγ 155, 156. We thus consider it necessary, that CVD studies targeting problems of transgenerational epigenetics induced or mediated by IGFs should use models where the IGF activity can be read in the context of the entire IGF‐system.

### Animal models for CVD based on metabolic determinants related to IGF‐ biology

To further evaluate the global and single nutrient deficiencies and their epigenetic basis as related to IGFs and CVD, we identified certain animal models that can be very useful to this matter. These prototypes are:

#### High‐fat diet in mice

This model resulted in an epigenetic phenotype with increased body weight, insulin resistance, hyperlipidaemia, hyperleptinaemia, and hypoadiponectinaemia 120, 121, 134, 135. The insulin resistance feature may involve IGFs compensatory activity in cell growth.

#### Maternal obesity in mice

‘Western Style’‐based diet induces maternal obesity and diabetes which allowed a phenotype with metabolic dysfunction and altered DNA methylation patterns in the mouse offspring 135, 136. The affected genes were more responsible for development than metabolic pathways. This change in foetal development leads to diabetes and obesity in the adult offspring 134, 135, 136, 137, 138, 139 and it might be related to IGFs and CVD.

#### Maternal hyperglycemia

In this case, an epipolymorphism was identified in placental leptin and adiponectin genes linking glucose metabolism to the maintenance of these genes. As mentioned earlier in this review, the cohort of patients born during the Dutch Hunger Winter, exposed to famine during gestation, show timing‐ and gender‐specific variations of methylation in a number of genes involved in metabolic control 113. These genes are responsible for glucose and lipid metabolism and consequently coronary artery disease in the adults which may be IGF‐dependent.

#### Ovine maternal obesity

The model shows a significant increase in hepatic expression of miR‐29b, miR‐103 and miR‐107 and a decrease in IR, phopsho‐AKT and phospho‐FoxO1 levels in the first generation of its offspring 140. In this case IGFs can play a major compensatory role.

#### Diet‐induced paternal obesity in mice

Molecular analyses demonstrated that diet‐induced paternal obesity in mice is associated with alteration of 414 mRNAs in the testis and 11 microRNAs in the sperm and all were associated with 25% DNA‐methylation/total DNA isolated from germ cells 134, 141. Recently, the transgenerational effects of paternal obesity have been also demonstrated in humans 142. This specific paternal metabolic dysfunction is associated with hypomethylation of the imprinted IGF‐II gene (igf2), as tested in the DNA‐pull extracted from umbilical cord blood cells 143, 147, 148.

#### Maternal obesity

Maternal obesity and paternal high‐fat‐diets induce metabolic dysfunctions and β‐cell impairment in the first generation (F1) of female rats. This last model shows a phenotype characterized by obesity and glucose‐intolerance which might be involved with IGF‐biology 134, 145.

## Conclusion

Epigenetics induced by IGFs fluctuations are likely linked to cardiovascular medicine. To date, there are no significant reports on epigenetics as related to clinical practise or therapeutics designed for transgenerational CVD forms associated or not to endocrine factors such as IGFs. In contrast, in oncology, epigenetic inhibitors have already shown curative potential 156. Understanding the epigenetic mechanisms induced by growth factors such as IGFs, gives assurance of significant contribution to understanding CVD in both paediatric and geriatric patients.

## Conflicts of interest

The authors have no conflict of interest to declare at this time.
